# E-assessment and an e-training program among elderly care staff lacking formal competence: results of a mixed-methods intervention study

**DOI:** 10.1186/s12913-015-0843-y

**Published:** 2015-05-06

**Authors:** Annika Nilsson, Maria Engström

**Affiliations:** Faculty of Health and Occupational Studies, Department of Health and Caring Sciences, University of Gävle, Gävle, Sweden; Department of Public Health and Caring Sciences, Section of Caring Sciences, Uppsala University, Uppsala, Sweden

**Keywords:** Competence in elderly care, E-assessment of prior learning, Information and communication technology, Informal education, Mixed-methods, Well-being, Working life

## Abstract

**Background:**

Among staff working in elderly care, a considerable proportion lack formal competence for their work. Lack of formal competence, in turn, has been linked to higher staff ratings of stress symptoms, sleep disturbances and workload. Objectives: 1) To describe the strengths and weaknesses of an e-assessment and subsequent e-training program used among elderly care staff who lack formal competence and 2) to study the effects of an e-training program on staff members’ working life (quality of care and psychological and structural empowerment) and well-being (job satisfaction and psychosomatic health). The hypothesis was that staff who had completed the e-assessment and the e-training program would rate greater improvements in working life and well-being than would staff who had only participated in the e-assessments.

**Methods:**

An intervention study with a mixed-methods approach using quantitative (2010–2011) and qualitative data (2011) was conducted in Swedish elderly care. Participants included a total of 41 staff members. To describe the strengths and weaknesses of the e-assessment and the e-training program, qualitative data were gathered using semi-structured interviews together with a study-specific questionnaire. To study the effects of the intervention, quantitative data were collected using questionnaires on: job satisfaction, psychosomatic health, psychological empowerment, structural empowerment and quality of care in an intervention and a comparison group.

**Results:**

Staff who completed the e-assessments and the e-training program primarily experienced strengths associated with this approach. The results were also in line with our hypotheses: Staff who completed the e-assessment and the e-training program rated improvements in their working life and well-being.

**Conclusion:**

Use of the e-assessments and e-training program employed in the present study could be one way to support elderly care staff who lack formal education by increasing their competence; increased competence, in turn, could improve their self-confidence, working life, and well-being.

## Introduction

Today, there is a need for more qualified staff in elderly care [1-3]. In Europe [2] and in countries such as Japan and the USA (US) [1], there is great concern about the growing proportion of older people [1-3]. An additional and important factor is retaining staff, as a large number of older people have multiple illnesses and complex care needs [4]. Staff working with older people are often nursing assistants and nurses with relatively low levels of education or no formal education at all [1,2]. Many of the staff are self-taught, thus skills have been acquired through informal learning [1,2]. Lacking formal competence has also been linked to greater sleep disturbances and higher workload [5]. Therefore, it is important to validate staff members’ competence and give them opportunities to develop and improve their knowledge, skills and ability. One way to develop staff members’ competence is with e-learning, which can be defined in different ways [6,7]. In the present context, e-learning refers to e-assessment of knowledge, skills and abilities and an e-training program supported by information and communication technology (ICT). The e-assessments in the present study are supported by ICT and were developed through a user-centered approach, where both trained staff and qualified teachers were involved throughout the process [8-10]. The assessment environment is adapted to reality, thus allowing teachers to capture different learning styles and base the assessment on the individual’s needs. However, the e-assessments are described in detail in another study [11].

## Background

In Sweden in 2011, approximately 213,024 people were working in municipal elderly care and the largest professional group included nursing assistants and licensed practical nurses [3,12]. Seventy-nine percent had a formal education, while the proportion across municipalities varied from 35 to 100 percent [3]. In other countries, the proportion of staff with no formal education has also been shown to be high [13], and in the US there is a shortage of nursing assistants [14]. It is difficult to recruit and keep young workers because the profession is often seen as being low in status [15].

Assessing non-formal and informal learning and providing competence development among staff working in elderly care may be seen as a way of improving lifelong learning. European countries stress the importance of prioritizing lifelong learning in education [16], where ICT is one method of providing education across the life span [17]. E-learning is often integrated into education and training among nursing students and educated health-care professionals [18,19], but studies employing e-learning among staff with no formal education are limited in number. However, several systematics reviews have shown that e-learning has increased participants’ knowledge [18,19]. Studies have also found no differences between e-learning and traditional learning with respect to knowledge, skills and satisfaction among nurses and student nurses [20]. Another literature review [21] among nursing students found that it is important to have ongoing education and support of nursing informatics, because such support helps students progress and gives them lifelong learning skills. Staff with no formal education in elderly care are often employed on a temporary basis owing to a staff shortage, which puts a strain on both the organization and the permanent staff [5]. Using e-learning among elderly care staff with no formal education allows valuable learning to occur outside formal education and training institutions (at work) [22]; it also highlights tacit knowledge and enables flexibility [18].

One model of healthy workplaces is the Practices for the Achievement of Total Health (PATH) model [23,24]. The PATH model has five categories of organizational practices that are thought to lead to healthy workplaces: work-life balance, employee growth and development, health and safety, recognition and employee development. The strategies lead to organizational improvements in two ways. First, the proposed organizational practices are supposed to directly lead to organizational improvements; second, the practices are supposed have an indirect impact on employees’ engagement, satisfaction, health and well-being, which, in turn, leads to organizational improvements. Studies have found that job satisfaction and motivation increase if staff are continuously able to: develop their competence and decide autonomously how they want to perform their job, feel satisfied when they receive organizational support, influence their work schedule and feel that their salary matches their competence [25,26]. Cross-sectional research has also shown relationships between structural conditions such as opportunities to learn and grow and higher staff ratings of job satisfaction, quality of care [5], skills and willingness to work according to evidence-based practice [27]. Having competence in one’s work is important, as it improves opportunities to control work-related situations [28].

The intervention under study here was designed to assess staff members’ knowledge and abilities, i.e. their established knowledge, skills and abilities learned over years of professional work in elderly care, as well as their needs for competence development. The intervention included both practical and theoretical e-assessments and a subsequent e-training program supported by ICT. Research has found that elderly care staff who lack formal education experience more stress symptoms and sleep disturbances [5]. Therefore, it is important that staff who lack formal skills be made visible in the organization and have better access to learning and growth opportunities and competence development via different pathways outside of and in addition to formal education. Having well-educated, experienced and competent staff is crucial to the provision of safe health care and to staff members’ well-being.

### Aims and the hypothesis

The aim was twofold: 1) to describe the strengths and weaknesses of e-assessments and subsequent e-training program used among elderly care staff who lack formal competence and 2) to study the effects of the e-training program on staff members’ working life (quality of care, and psychological and structural empowerment) and well-being (job satisfaction and psychosomatic health). The hypothesis was that staff who had completed both the e-assessments and the subsequent e-training program would rate greater improvements in their working life and well-being than would staff who had only participated in the e-assessments.

## Methods

### Design

An intervention study was conducted using a mixed-methods approach with quantitative (2010–2011) as well as qualitative data (2011) [29].

### Setting and procedure

In central Sweden, one municipality, which provides healthcare and general services in residential homes and in-home care for older people, was involved in the project from 2010 to 2011. In 2009 an inventory of formal education in care was completed by all staff members (n = 887) and became the basis for the e-assessment to 89 staff in relation to their job requirements. Of them 87 underwent both the practical and the theoretical e-assessments. The e-assessments were voluntary and offered by the manager via personal meetings and email. The competence needs (i.e., deficient knowledge and skills) identified at the individual level in the e-assessments were the basis for implementation of a professional e-training program tailored to every individual’s needs. The e-assessments – three practical and eight theoretical e-assessments of staff members’ knowledge, skills and abilities – as well as the completed education were based on the curriculum of the Swedish upper secondary school care program, which includes a total of eight learning objectives [30].

### E-assessments and the subsequent e-training program

*Three practical assessments –* morning, lunch and evening – were conducted in a specifically designed apartment with video cameras and ICT technology. They included a total of 5 learning objectives with different grades and were related to everyday work: “Basic health care”, “Communication”, “Rehabilitation”, “Ergonomics, hygiene, esthetics, environment” and “Assistive technology”. The practical skills assessments were carried out by creating everyday situations and took about 40 minutes/person/situation. One test leader was involved, a qualified teacher working in the national care program, who supervised the practical assessments on the computer via video transmission, with both picture and audio transfer. The test leader also judged the staff members based on strict checkpoints and guidelines for the situation. The check points and guidelines were based on the curriculum, goals and grading criteria for the Swedish national care program’s first course “Healthcare and Social Work” and are established by the Swedish National Agency for Education [30].

*The theoretical assessments* were performed using a workplace-based ICT tool (computer) that consisted of 8 learning objectives: “Health”, “Communications”, “Oral care”, “Ergonomics, hygiene, esthetic, environmental”, “Rehabilitation”, “Assistive technology”, “Basic health care”, and “Law and organization”. The items were arranged in order, from those expected to be easiest to those expected to be more difficult. The pedagogical aim was to highlight the individual’s strengths and self-esteem. The assessments took about 30 minutes/person/situation.

After completion of the e-assessments, staff received a document accredited by the Center for Adult Education in the municipality that was to be used for the individual’s future competence development. The subsequent e-training program was individualized and linked to staff members’ practical and theoretical e-assessment outcomes as well as tailored to suite their learning styles. The e-training program began with the teacher giving an individual follow-up, a debriefing, in relation to the completed e-assessment. After that, the teacher helped the staff member tailor his/her competence development and he/she began studying the areas of deficiency. When the staff member felt he/she was ready to take the practical and/or theoretical test/tests, which were the same as in the e-assessments, he/she was to contact the teacher. The theoretical part was performed through an ICT tool that could be used either at the workplace or at home. This meant that the e-training program could be carried out independent of time and place and be done all at once or divided up. This, of course, depended on how many e-assessments the staff members had failed on. The range of number of failures for the theoretical e-assessments was 2–8, and for the practical e-assessments 1–3. As the e-training program was individualized, it took various lengths of time (1 day to 6 months) for the staff members to complete it. ICT supervision and support were given to the individual by the teacher, whose role was also to serve as a coach when the individual felt the need for guidance and/or support.

### Sample

**Figure 1:**
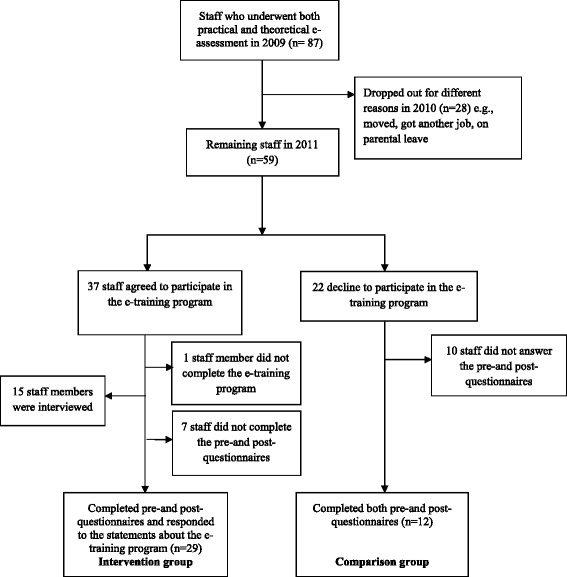
Flow chart illustrating the study sample and dropouts.

Of the 87 staff members with no formal education who had completed the e-assessments, 59 remained in 2011 when the e-training program was to be started. During 2010, 28 staff members had dropped out for different reasons (e.g., moved, got another job, on parental leave or retired). All eligible staff (n = 59) were invited to take part in the e-training program. In 2011, 37 of the 59 staff began the e-training program and, of these, 36 completed it. Twenty-two of the staff members declined to participate in the e-training program. Several of them were not interested, while others felt they were too old. Of the 36 who completed the e-training program, 15 were interviewed. Purposive sampling was used to ensure variation in staff members’ age, gender and e-assessment results. Of the 36 staff who completed the e-training program, 29 had completed both the pre- and post- questionnaires (see data collection) and had responded to the statements about the e-training program. Two staff members only responded to the statements about the e-training program (also interviewed). Those who did not participate in the e-training program, but who completed the questionnaires on both occasions, comprised the comparison group (n = 12) (see Figure 1).

### Data collections

Data were collected through interviews and questionnaires. Staff members’ opinions about the subsequent ICT-supported training program were measured using 10 statements in a *study-specific questionnaire,* with response alternatives on a 5-point scale. The statements and semi-structured interviews were inspired by a study based on Roger’s diffusion of innovation theory [31,32]. An interview guide (see Appendix 1) comprising open-ended questions was used [33,34]: “I want you to think back to the first time you heard about e-assessments, what were your first thoughts?”, “Based on these, talk about your first thoughts concerning how you felt before, during and after the e-assessments”. “How did you feel when you were sitting outside the training apartment and what were your thoughts in the apartment and afterwards?”, “What was it like to perform the theoretical tests using a computer?”

Examples of questions about the e-training program were: “I want you to think back to the first time you heard about the e-training program, what were your first thoughts?”, “Based on these, talk about your first thoughts concerning how you felt before, during and after the e-training program?” Questions concerning perceived usefulness and ease of use were also asked: “If you compare the e-training program you have completed now to a traditional education, what do you feel are the pros and cons?”, “If you think about the ICT, was it difficult/easy to use?”, “What is your previous experience of ICT?” Probes were used to obtain richer descriptions. The interviews were carried out at the staff members’ workplaces by the first author; they lasted 25–30 minutes and were tape-recorded and transcribed verbatim. The questionnaires below were administrated to the staff members before and directly after they had performed the e-training program (intervention group); the same time interval was used for the comparison group.

Job satisfaction, quality of care and psychosomatic health were measured using *The Satisfaction with Work Questionnaires (SWQ)* [35]. The Job Satisfaction scale consists of 49 items measuring eight factors: personal development (α-value in the present study was 0.79), workload (α 0.84), criticism (α 0.85), expectations and demands (α 0.85), cooperation (α 0.85), internal motivation (α 0.85), external motivation (α 0.86) and position in the group (α 0.85). The Quality of Care scale consists of 24 items measuring four factors: nursing and medical care (α 0.85), documentation (α 0.87), communication ability (α 0.85) and communication obstacle (α 0.85). The Psychosomatic Health scale consists of 19 items measuring two factors: sleep (α 0.85) and stress symptoms (α 0.84). For all three scales, the response alternatives are presented on a 5-point scale, and a higher value represents a more desirable state. Total scores and factor scores are transformed to values between 0 and 100. The psychometric properties of the three scales have been reported to be acceptable [35].

Structural empowerment was measured using *The Conditions of Work Effectiveness Questionnaire II (CWEQ II)* [36], which was translated to Swedish by Engström et al. [5]. The instrument consists of 19 items measuring six components of structural empowerment: information (α 0.86), opportunity (α 0.86), support (α 0.86), resources (α 0. 87), informal power (α 0.86) and formal power (α 0.86). Response alternatives are presented on a 5-point Likert-type scale for all items. Factor scores are averaged, and the total score for the instrument (total empowerment) is the sum of the factor scores. Higher scores represent a higher perception of structural empowerment. Cronbach’s Alpha values for the factors in CWEQ II have been reported to exceed 0.70 [37,38]. Construct validity has been tested and reported to be satisfactory/good [36]; this applies to the Swedish version as well [5].

*Spreitzer’s Empowerment Scale* [39] has been translated to Swedish by Hochwälder and Bergsten Brucefors [40] and is used to measure psychological empowerment. The scale consists of 12 items (α 0.81) measuring four factors: meaning (α 0.86), competence (α 0.86), self-determination (α 0.86) and impact (α 0.86). The response alternatives are presented on a 7-point Likert scale; the total score and factor scores are averaged to form indexes that range from 1 to 7, where higher scores represent higher perceived psychological empowerment. Psychometric properties of the Swedish version have been reported to be satisfactory [40]. A second-order factor analysis has shown an acceptable fit between the data and the theoretical model. Cronbach’s Alpha values for the factors varied between 0.77 and 0.90 [40].

### Data analysis

The interview data were analyzed using qualitative content analysis [33,34]. The interviews were listened to and the text was read through several times. Text related to the first part of the aim was identified as meaning units, condensed and then abstracted and labeled with a code. Codes were then compared for differences and similarities and sorted into categories. The two researchers (AN and ME) reviewed some of the interviews together, and then developed codes, subcategories and categories. Questionnaire data: Mann–Whitney-U test was used to compare differences over time (year 2010 and 2011) between the intervention and comparison group. Wilcoxon signed-rank test was used to compare differences over time within the intervention and the comparison group. Internal consistency was measured for all instruments using Cronbach’s Alpha. All analyses were conducted with IBM statistics SPSS 20. The level of statistical significance was set at p < 0.05 (two-tailed).

### Ethical considerations

The Central Ethical Review Board in Uppsala approved the study (reg. no. 2009/430). The study was also approved by the head of operations for elderly care in the municipality. The study participants received written information about the study, were informed that participation was voluntary and were ensured confidentiality.

## Results

### Staff characteristics

In total, 41 staff participated in the study, and of them 15 (n = 12 female) were interviewed. The mean age was 42 years (SD 9.4, 26–60), and number of years working in elderly care ranged from 6 to 28 (mean number of years = 10.6, SD 7.4). In all, 41 staff were included in the quantitative analysis: 29 in the intervention group; 25 women and 4 men ranging in age from 22 to 54 years. Number of years working in elderly care varied between 4 and 32. The comparison group included 12 staff: 11 women and 1 man, ranging in age from 25 to 57 years. Number of years working in elderly care varied between 7 and 34. The intervention and comparison groups’ characteristics are presented in Table 1. No statistically significance differences were found between the groups’ characteristics and the study variables in 2010.

**Table 1:** **Staff characteristics (n = 41)**

**Characteristics**	**Intervention group (n = 29)**	**Comparison group (n = 12)**
Women, n (%)	25 ( 86)	11 ( 92)
Age, mean, (SD)	42 (10.6)	45 (10.5)
Years working in elderly care, mean (SD)	10 (6.4)	14 (7.8)

### Staff members’ experiences of the strengths and weaknesses of the e-assessments and the subsequent e-training program

Analysis of staff members’ reflections on the e-assessments and subsequent e-training program resulted in two main categories: “Obstacles/Barriers” and “Enablers”, each of which were derived from three sub-categories. Each category and its subcategories are presented separately. Under the respective headings, the content of each category is described and illustrated using staff members’ statements, which are inserted into the text and marked with quotation marks and italics.

#### Obstacles/Barriers

The category *Obstacles/Barriers* consisted of three subcategories representing staff descriptions of *Being nervous*, *Not having confidence in their own abilities,* and *Internal and external demands*. Such descriptions were particularly common in relation to the practical e-assessments carried out in a specifically designed apartment equipped with ICT, including video-recording. However, several of the staff also described being nervous about the subsequent e-training program. No one in the staff passed all of the assessments.

#### Being nervous

Before starting the practical e-assessments and the e-training program, most of the staff described being nervous about what was to come and what was expected of them. Concerning the practical e-assessments (morning, lunch and evening assessments), some of the staff were nervous about being observed by the cameras set up in every room: *“Before it I was nervous about being filmed from all angles.... I was actually nervous…”* (Informant 4). Others said that it was *“… mostly the first morning part… and then once you’re in there and have started ‘yeah, yeah, the cameras’ you thought after a couple minutes, but then you felt like no I’ve forgotten them… I didn’t think about them”* (Informant 3). Several of the staff described the morning assessment as “artificial” because they had to work with an adult-sized doll, which was *“so different, it was like what should I do what should I say and talking yes it was more difficult to talk in a natural way…”* (Informant 5). Lunch and evening assessments were experienced as more real because there they worked with a person posing as an older care recipient. *“Couldn’t find things in the apartment but that’s what it’s like when you’re in a client’s home. The actor was true to reality”* (Informant 5). These situations were more realistic and “everyday-like”. Concerning the theoretical assessments, none of the staff reported being nervous, though some were concerned that there would be a great deal to read*. “It was fun but I was a bit worried there’d be a lot to read”* (Informant 2).

#### A sense of uncertainty

Several of the staff placed high demands on themselves regarding both the practical and theoretical assessments, while some were surprised about not having passed more tests than they did. Concerning the practical assessments, especially the morning assessment, they felt that it was difficult to know what to say or talk about. The situation in itself made it easy to make mistakes, which could be interpreted as them being unable to do their work : *“… mostly if I made a mistake and then I thought God if I do something wrong here it means I don’t know my job, that’s how it felt because really I’m supposed to know this”* (Informant 2). In the apartment, several of the staff felt they spent a great deal of their energy keeping an extra sharp eye on where things were. “*It felt like meeting a new pensioner.... but I know what I know and I know what to do during the morning work”* (Informant 6). Regarding the theoretical assessments, some of the staff found them troublesome, because they felt that some of the issues were invented, which made them uncertain about passing the tests. Issues related to oral care and hearing aids were seen as odd, because some of the staff had no knowledge in these areas. Others said that the theoretical assessments were not in concordance with how they worked every day: *“I thought the questions on the computer were difficult, well I thought so, you work in a certain way… yeah, it was a bit difficult”* (Informant 10).

#### Internal and external demands

Prior to the e-assessment and subsequent e-training program, some of the staff experienced negative feelings, such as not being able to cope with the challenge and being afraid of misinterpreting the information: *“ … can I manage this, I really don’t have a head for studying”, “.... you don’t think you can do things, but that’s how it is, you can do more than you think”* (Informant 11). One staff member said she felt her employer made demands on her. She thought it could depend on a previous occasion when she was dismissed due to cutbacks. She was later re-employed and felt motivated to continue the training program though she was not sure she could manage it. *“But I feel that the municipality is demanding this of me and I want a job. And I might not end up with a job. I was fired once two years ago. But that wasn’t because I don’t have the right education but because of cutbacks”* (Informant 7). Concerning the theoretical assessments, some of the staff experienced pressure because there was a time limit and lack of information and support from the teacher. At the same time, they felt they were motivated to continue the e-training program despite feelings of uncertainty. Regarding the e-training program, some of the staff experienced time pressure and placed great demands on themselves. *“Not much gets into my brain, I have so much other crap in there, there’s no room for more and it doesn’t matter how much I read about things they just don’t stick”* (Informant 9). Although some of the staff felt these kinds of demands both before and during the e-training program, most of them completed it. As can be seen in Table 2, the majority of the staff felt the e-training program was good.

**Table 2:** **Staff members’ ratings of the e-training program (n = 31)**

**Statements about the e-training program**	**Totally disagree**	**Partly disagree**	**Neither agree nor disagree**	**Partly agree**	**Totally agree**
1. I feel that the content of the e-training program was good, i.e., adapted to my needs.			1	15	15
2. I feel that the e-training program was well conducted.		1	2	7	21
3. Communication between me and the teacher has been good.				4	27
4. In my view, my ability to manage the IT*-based part of the training program (the computer) has been good.			1	9	21
5. I feel that the IT*-based training program has made me more independent, in that I’ve been able to manage on my own.		1	1	6	23
6. I think that the IT*-tool has been a good aid to learning.			2	3	26
7. I needed the help of another person every time I used the IT* tool.	23	7	1		
8. I’m used to using the IT* tool		1	4	13	13
9. I would recommend this study method to my colleagues.			1	4	26
10. If other e-training programs were available using this method I would choose them over traditional courses.			3	4	24

IT* = Information technology.

### Enablers

The category *Enablers* consists of three subcategories: *Having opportunities to develop in a flexible and easily accessible way, Becoming aware and being able to influence* and *Feeling stronger.* The staff described this category in terms of being able to test their skills and knowledge in an easy, flexible and accessible way as well as feeling motivated to receive grades, seeing possibilities and experiencing that their self-confidence increased during the process.

#### Having opportunities to develop in a flexible and easily accessible way

When staff heard about the e-assessment and the subsequent e-training program, their first thoughts were positive. They saw it as an opportunity to highlight their skills and knowledge in an easy and accessible way, especially when the employer wanted it: *“Great, they presented it really well, and there were a lot of us who thought finally… because they were anxious for us to do it. That made it more fun too”* (Informant 3). Most of the staff were motivated to take part in the assessments and the e-training program as a way to highlight their skills and knowledge using ICT. This was also shown in the study-specific questionnaire (Table 2) with regard to the e-training program, in that most of the staff thought that it was good to take the theoretical tests on the computer (n = 23 totally agree, n = 3 partly agree). In the interview, several of the staff said they appreciated that *“…you got feedback right away so you knew what you’d done wrong”* (Informant 1). Concerning the program using ICT, the teacher could give feedback directly and staff could carry out the e-training program (tests) at their own pace. These results corresponded to the study-specific statements, where nearly all agreed that the program was tailored to meet individual needs (n = 15 totally agree, n = 15 partly agree), that the implementation was good (n = 21 totally agree, n = 7 partly agree) and that the communication between the teacher and staff had been very good (n = 27 totally agree, n = 4 partly agree).

#### Becoming aware and being able to influence

After the e-assessments and subsequent e-training program, the majority of staff experienced increased awareness of what they were doing at work, especially *“why they do it and what they do it for”*. In their daily work, the new knowledge made them think differently: *“I think more now, when I’m working, about what I’m doing”* (Informant 5). They were also more aware of how their colleagues worked: *“… now I see how my workmates are stuck in their patterns, and so I can, if you have newer information about what you can do at work”* (Informant 5). In some cases, staff reported having transferred what they had learned to their workplaces: *“good that there were also changes at the workplace”* (Informant 3) and that they could now also discuss *“this new knowledge”* with their workmates. Several of the staff felt it was a great opportunity to test their skills and knowledge, so as to have some influence on their work. If they wanted to advance at work in nursing, they must have some training. However, others said that they had thought about educating themselves, but felt they had too much catching up to do to get a degree. Still, several said they wanted to continue studying to become licensed practical nurses, if they could do it in the same way as in the e-training program *“really it makes me want to continue, it does. Well I do hope they do more of this… so that everyone who wants to can keep learning all the licensed practical nurse material this way”* (Informant 3). These results corresponded to responses on the study-specific questionnaire, where most of the staff (n = 24 totally agree, n = 4 partly agree) were willing to continue taking part in the assessments and training program supported by ICT, while only 3 staff members neither agreed nor disagreed (Table 2).

### Feeling stronger

In this subcategory, staff described being proud and having trust in their own abilities. Several were happy and proud that they had passed the e-training program: *“… I was proud of myself, never thought it could have gone as well as it did… I’ve always had difficulties with tests”* (Informant 9). *“I’m glad I did it, I’m done, the fact that I could do it at all, what I didn’t think I could”* (Informant 10). They said that their independence and self-confidence increased during the process and that they felt stronger: *“… I can manage myself to a greater degree, and I mean if you need help there is a back-up”* (Informant 3). Results from the interviews are also consistent with responses to the study-specific questionnaire statements: “I feel that the IT-based training program has made me more independent, in that I’ve been able to manage on my own” (n = 23 totally agree, n = 6 partly agree, Table 2). Most of the staff said that the e-training program was well organized and that the teacher was easily accessible and gave quick/immediate feedback. That was perceived as a source of security, which meant that the staff dared to expose themselves to what could be perceived as difficult: *“… I told her that I didn’t have a head for studying, that I didn’t know anything, and she said you can come to me so we’ll see what I can help you with … I’m pleased with myself now”* (Informant 11).

### Staff members’ estimation of psychosomatic health, job satisfaction and quality of care

The interviews revealed that the staff felt stronger, and the quantitative data over time support these results. Staff in the intervention group (n = 28) rated their psychosomatic health as better over time (total scale for 2010 Mean = 18.5, SD = 2.6 vs. for 2011 Mean = 19.5, SD = 2.6; p = 0.040) as well as the factor “sleep problems” (2010 Mean = 59.8, SD = 25.4 vs. 2011 Mean = 67.0, SD = 25.3; p = 0.036). For both the factor and total scale, higher scores mean fewer health problems. In the comparison group, staff rated their sleep significantly worse over time (2010 Mean = 59.7, SD = 31.4 vs. 2011 Mean = 48.6, SD = 25.4; p = 0.034; n = 12). Staff in the intervention group also rated their job satisfaction as better over time for the factors “personal development” (2010 Mean = 65.6, SD = 13.0 vs. 2011 Mean = 71.6, SD = 10.3; p = 0.031) and “position in the group” (2010 Mean = 56.0, SD = 13.1 vs. 2011 Mean = 59.5, SD = 13.2; p = 0.048). Furthermore, they rated quality of care as better over time; total score of quality of care (2010 Mean = 69.7, SD = 9.8 vs. 2011 Mean = 74.6, SD = 8.4; p = 0.029; n = 27). When differences over time were compared between the groups, there were significant differences for the factor sleep (intervention group Mean differences = 7.1, SD = 17.8 vs. comparison group Mean differences = −11.1, SD = 14.8; p = 0.003). No other significant differences were found between the groups when comparing differences over time.

#### Staff members’ estimation of psychological empowerment and structural empowerment

Staff in the intervention group gave significantly higher scores, over time, for the factor “impact” on the psychological empowerment scale (2010 Mean = 4.4, SD = 1.3 vs. 2011 Mean = 5.1 SD = 0.8; p = 0.005; n = 26). Furthermore, they rated better structural empowerment over time (total score 2010 Mean = 18.5 SD = 2.6 vs. 2011 Mean = 19.5, SD = 2.7: p = 0.008: n = 26), as well as for the factors “formal power” (2010 Mean = 3.0, SD = 0.7 vs. 2011 Mean = 3.3, SD = 0.8: p = 0.022) and “informal power” (2010 Mean = 2.9, SD = 0.6 vs. 2011 Mean = 3.2, SD = 0.6: p = 0.011). When differences over time were compared between the groups, there were significant differences for the scale Psychological empowerment (intervention group Mean differences = 0.3, SD = 0.6; n = 26 vs. comparison group Mean differences = −0.2, SD = 0.7; p = 0.033) as well as for the factors “self-determination” (intervention group Mean differences = 0.3, SD = 1.0; n = 26 vs. comparison group Mean differences = −0.4, SD = 1.4; p = 0.042) and “impact” (intervention group Mean differences = 0.7, SD = 1.0; n = 26 vs. comparison group Mean differences = −0.4, SD = 0.8; p = 0.003). Regarding Structural empowerment, no significant differences were found over time between the groups.

## Discussion

The present study showed that most of the staff who completed the e-assessments and the subsequent e-training program in elderly care primarily experienced strengths associated with this approach. Several of the staff would also recommend this approach to colleagues. If additional education/training could be performed in this way instead of traditional education, most of the staff would choose it. However, before and during the e-assessments and the e-training program, some of the staff expressed that they were nervous and that they did not have confidence in their own abilities. They also experienced internal demands from themselves and external demands from the employer, workmates and family, which sometimes made it difficult to cope with the challenge. During and after conducting the e-training program, staff talked about how their self-confidence increased. The interviews revealed that staff felt stronger and the quantitative data over time supported these results, which were also in line with our hypotheses, i.e. that staff who had completed the e-training program would rate greater improvement in working life and well-being than would staff who had only participated in the e-assessments.

To our knowledge, this is the first study to measure working life and well-being outcomes following e-assessments and an e-training program intervention among elderly care staff lacking in formal education, and where results support the PATH model. The PATH model [23,24] indicates that five categories of organizational practices lead to two paths toward organizational improvement (where two of these five – employee growth and development and recognition – were included in the present study). According to the PATH model, these strategies lead both directly and indirectly to organizational improvements such as lower absenteeism, lower employee turnover, and higher quality of work. In the present study, the staff members reported having opportunities to develop in a flexible and easily accessible way, becoming aware and being able to influence their workplace; moreover, after the intervention they gave higher ratings for their impact as well as quality of care. The indirect way in which the strategies lead to organizational improvements, according to the PATH model, is through employees’ improved well-being, including better physical and mental health, improved job satisfaction and motivation. Our results confirm that two of the strategies in the PATH model – employee growth and development and recognition – had an impact on job satisfaction, psychosomatic health and empowerment in the intervention group. The intervention group rated their psychosomatic health as better over time and showed improvements in personal development and position in the group. Concerning psychological and structural empowerment, the intervention group gave higher scores over time for the factor “impact” in the psychological empowerment scale. This factor concerns the degree to which a person can influence strategic, administrative, or operating outcomes at work [39]. The interviews showed that, for some of the staff, this new knowledge made them think differently and become aware of what they were doing and why they did it in the context of their daily work. Some of them had also transferred what they had learned at their workplace; they experienced that they now have some influence on their work. The intervention group also rated better structural empowerment over time. The interviewees who had completed the e-assessments and the e-training program reported how their self-confidence had improved during the process.

A review [41] of which intended effect education/training programs have on staff working in dementia care concluded that the education/training program should be based on staff needs, as identified by staff members themselves. This is in line with the inclusion criteria for the current study, where all staff lacking formal education were invited, and they thereafter reported voluntarily their interest in participating in competence development. Furthermore, the e-training program was individualized and based on each staff member’s practical and theoretical assessment outcomes as well as tailored to suite their learning styles. The study-specific questionnaire and interviews confirm that most of the staff members felt the content of the e-training program and the way in which it was carried out were appropriate to their needs. According to Eraut and Hirsh [42], assessment of learning style is central to any training program.

Studies [41] have shown that the organization should capture staff members’ own motivation and promote conditions for adult learning (i.e. self-study, experiential learning) as well as that the work climate, work organization and the nature of management support affect whether staff training will lead to competence development [43,44]. Having a supportive work environment and competence in elderly care have been found to be important to caregivers’ ability to manage their work and to supporting a feeling of security in their professional role. A previous interview study also found that a supportive work environment and competence resulted in increased commitment to work and professional development [45]. Our results also confirm earlier cross-sectional studies showing that opportunities to learn and grow are linked to higher staff ratings of job satisfaction, quality of care [5], skills and willingness to work according to evidence-based practice [27]. Huskanen and Graue [46], who also used mixed methods, found that support and willingness on the part of managers in the municipality are important in encouraging staff members to participate in training programs. In the current study, some staff members reported being motivated when managers in the municipality encouraged those with no formal education to take part in the competence development program. Staff members who participated in the e-training program were also given extra vacation days.

In summary, staff who had completed both the e-assessments and the subsequent e-training program rated greater improvements in their working life and well-being than did staff who had only completed the e-assessments. The results support the PATH model. Assessing and evaluating informal learning, such as knowledge, skills and abilities, and thereafter offering an e-training program based on assessment results, may be a way of improving lifelong learning.

The present study has some limitations that need to be considered. Its non-randomized design might have induced selection biases, and the small sample size and high drop-out rate limit generalizability. Another limitation is that a test of inter-rater reliability regarding the e-assessment tool was not performed in this study and that the sample was taken from only one municipality in Sweden. Workplace culture may have played a role in how the individuals felt about the e-training program and may limit generalizability. However, staff members in the intervention group did not differ from those in the comparison group in sociodemographic characteristics and the study variables at baseline. Strengths were the use of a mixed-method design and of validated instruments with satisfactory internal consistency; in the present study, Cranach’s Alpha for all factors and total scores were <0.70. However, to our knowledge, this is the first study to measure working life and well-being outcomes following e-assessments and an e-training program intervention among elderly care staff lacking in formal education. Future studies should test e-assessments and e-training programs using larger samples sizes and in different areas of health care.

## Conclusion

The present e-assessments and the e-training program could be one way of helping elderly care staff who lack formal education develop their competence, which would, in turn, improve their self-confidence, working life and well-being. Thus, the e-assessments and the e-training program lead, in accordance with the PATH model, to healthy workplaces and organizational improvements. In order to increase quality and security in elderly care, it is important to test different pathways when identifying needs for competence development among staff and to give them opportunities to learn and develop through training.

## Appendix 1

### Interview guide

#### E-assessments

I want you to think back to the first time you heard about e-assessments, what were your first thoughts?

Based on these, talk about your first thoughts concerning how you felt before, during and after the e-assessments.

How did you feel when you were sitting outside the training apartment and what were your thoughts in the apartment and afterwards?

What was it like to perform the theoretical tests using a computer?

Is there anything you would like to add?

### E-training program

I want you to think back to the first time you heard about the e-training program, what were your first thoughts?

Based on these, talk about your first thoughts concerning how you felt before, during and after the e-training program?

If you compare the e-training program you have completed now to a traditional education, what do you feel are the pros and cons?

If you think about the ICT, was it difficult/easy to use?

What is your previous experience of ICT?”

Is there anything you would like to add?
